# Why Do Tetrapropylammonium Chloride and Sulphate Salts Destabilize the Native State of Globular Proteins?

**DOI:** 10.1155/2014/870106

**Published:** 2014-01-27

**Authors:** Giuseppe Graziano

**Affiliations:** Dipartimento di Scienze e Tecnologie, Università del Sannio, Via Port'Arsa 11, 82100 Benevento, Italy

## Abstract

It has recently been shown that aqueous solutions of tetrapropylammonium chloride and sulphate salts destabilize the folded conformation of Trp-peptides (Dempsey et al., 2011). This result is rationalized by the application of a statistical thermodynamic approach (Graziano, 2010). It is shown that the magnitude of the solvent-excluded volume effect, the main contribution for the native state stability, decreases in both aqueous 2 M TPACl solution and aqueous 1 M TPA_2_SO_4_ solution. This happens because TPA^+^ ions are so large in size and interact so weakly with water molecules, due to their very low charge density, to be able to counteract the electrostrictive effect of chloride and sulphate ions on the water structure, so that the density of their aqueous solutions is smaller or only slightly larger than that of water.

## 1. Introduction

It is becoming increasingly clear that complex ions can play an important role for the conformational stability of peptides and globular proteins. Recently, Dempsey, Mason, and Jungwirth, DMJ, have experimentally shown that the folded conformation of Trp-zipper peptides is destabilized by salts of the tetrapropylammonium, TPA^+^, ion [1]. In particular, destabilization occurs in both aqueous 2 M TPACl solution and aqueous 1 M TPA_2_SO_4_ solution. This result sounds somewhat unexpected because, in the case of the corresponding guanidinium salts, GdmCl proves to be a strong destabilizing agent, whereas Gdm_2_SO_4_ has no effect on the conformational stability of Trp-zipper peptides [1]. Note that, in general, GdmCl strongly destabilizes the native state of globular proteins, whereas Gdm_2_SO_4_ stabilizes the native state of globular proteins [2].

According to the rationalization proposed by DMJ [1, 3, 4], (1) TPA^+^ ions, like Gdm^+^ ions, should favourably interact with nonpolar side chains, causing the destabilization of native folded conformations; (2) sulphate ions form ion pairs with Gdm^+^ ions, reversing the denaturing activity of guanidinium ions; (3) sulphate ions are not able to form ion pairs with the very large TPA^+^ ions and so cannot reverse the denaturing activity of the latter.

I have developed a different explanation for the contrasting action of GdmCl and Gdm_2_SO_4_ towards the native state of globular proteins [5], by extending the statistical thermodynamic approach devised to explain the molecular origin of cold denaturation [6]. The fundamental quantity is the difference in the reversible work to create in aqueous solution a cavity suitable to host the denatured state and a cavity suitable to host the native state. In aqueous GdmCl solutions, this contribution is not large enough to overwhelm the conformational entropy gain upon unfolding and the direct attractions between Gdm^+^ ions and protein surface groups; in aqueous Gdm_2_SO_4_ solutions, it is so large that it overwhelms the two destabilizing contributions [5]. Sulphate ions, SO_4_^2−^, due to their high charge density, interact strongly with water molecules producing a number density increase, that, in turn, renders the cavity creation process very costly, reversing the denaturing activity of Gdm^+^ ions, and stabilizing the native state of globular proteins [5]. The reliability of this rationalization is confirmed by the experimental finding that sulphate ions do not form ion pairs with Gdm^+^ ions [7], in contrast to the DMJ claim. The same theoretical approach has provided a reliable explanation for the TMAO ability to counteract the denaturing activity of urea [8].

I have tried to further use the same approach to analyse the effect of TPACl and TPA_2_SO_4_ on the conformational stability of the native state, by using the experimental density value of aqueous 2 M TPACl solution [9] and a reliable estimate of the density of aqueous 1 M TPA_2_SO_4_ solution. It results that in both cases the difference in the reversible work to create a cavity suitable to host the denatured state and a cavity suitable to host the native state is smaller than that calculated in water, leading to a destabilization of the native state in line with experimental data. This confirms the general validity of the devised approach and the fundamental role played by the solvent-excluded volume effect for the conformational stability of globular proteins.

## 2. Theory Section

The basic ground of the theoretical approach is the recognition that the insertion of a solute molecule in all solvents produces a solvent-excluded volume [6]. The latter is not represented by the volume of the cavity that has to be created in the liquid to host the solute molecule because, by operating at constant pressure, the volume of the liquid will increase upon cavity creation by a quantity equal to the partial molar volume, PMV, of the cavity itself. The solvent-excluded volume is represented by the shell region between the van der Waals surface of the cavity and its solvent accessible surface. The centres of solvent molecules cannot enter this shell region because the cavity volume must be empty to allow the insertion of the solute molecule. The occurrence of such solvent-excluded volume causes a reduction of the configurational space available to solvent molecules and so a decrease in the configurational/translational entropy of solvent molecules. This entropy decrease is the molecular origin of the poor solubility of nonpolar species in water because its magnitude is larger in water than in the other common liquids mainly because of the small size of water molecules [10].

A polymeric solute can adopt very different conformations in a solvent producing different solvent-excluded volumes, even though the van der Waals volume of the chain, *V*_*vdW*_, does not change. In water these different solvent-excluded volumes are well captured by the values of the water accessible surface area, WASA, of the various conformations [5, 6]. The collapse of a hydrophobic polymer in water, at constant pressure and temperature, should mainly be driven by the minimization of the solvent-excluded volume (i.e., WASA minimization) in order to maximize the configurational/translational entropy of water molecules with the constraints assigned to the thermodynamic system (i.e., a hydrophobic chain in water). In the case of globular proteins in water and aqueous solutions, it is possible to divide all the available conformations in two macrostates: the D-state (i.e., the average structure of the ensemble of denatured conformations, whose properties are not affected by the presence of denaturing or stabilizing agents in aqueous solution) and the N-state (i.e., the average structure of the ensemble of native conformations). Complete development of the theoretical approach shows that the contribution always stabilizing the N-state is the difference in the reversible work to create in aqueous solution a cavity suitable to host the D-state and a cavity suitable to host the N-state [5, 6], ΔΔ*G*_*c*_ = Δ*G*_*c*_(D-state) − Δ*G*_*c*_(N-state). The latter term is the actual ground of what is usually called the hydrophobic effect. It is a purely entropic quantity [11], measuring the loss in configurational/translational entropy of water molecules for the increase in solvent-excluded volume associated with protein unfolding. The simple calculation of the magnitude of the ΔΔ*G*_*c*_ term in water, aqueous 2 M TPACl solution, and aqueous 1 M TPA_2_SO_4_ solution should allow the classification of these two salts as denaturing agents.

I have adopted the same calculation procedure devised to rationalize several aspects of the conformational stability of globular proteins [5, 6, 8]. I have assumed that (1) the N-state can be represented as a simple sphere, whereas the D-state can be represented as a prolate spherocylinder, possessing the same *V*_*vdW*_ of the sphere representing the N-state, but a markedly larger WASA; (2) specifically, the N-state is a sphere of radius *a* = 10 Å, *V*_*vdW*_ = 4189 Å^3^, and WASA = 1633 Å^2^ (calculated using for water molecules the customary radius of 1.4 Å), whereas the D-state is a prolate spherocylinder of radius *a* = 4 Å, cylindrical length *l* = 78 Å, *V*_*vdW*_ = 4189 Å^3^, and WASA = 3013 Å^2^; these numbers are reliable for a 50-residue globular protein; (3) the ΔΔ*G*_*c*_ term is estimated by calculating the reversible work to create in water and in aqueous salt solution the corresponding cavities, by assuming that (a) water can be treated as a hard sphere fluid possessing the experimental density of water at the desired temperature; (b) aqueous salt solutions can be treated as hard sphere fluid mixtures possessing the experimental density of real aqueous salt solutions at the desired temperature.

The classic scaled particle theory [12], SPT, formula for a spherocylindrical cavity of radius *a* and cylindrical length *l* in a hard sphere fluid mixture, derived by means of the geometric approach [5] (the pressure-volume term is neglected for its smallness at *P* = 1 atm), is

(1) ΔGc=RT·{−ln⁡(1−ξ3)+[6ξ2(1−ξ3)]a     +  [12ξ11−ξ3]a2+[18ξ22(1−ξ3)2]a2       +  [3ξ22(1−ξ3)]l+[6ξ1(1−ξ3)]a·l      +[9ξ22(1−ξ3)2]a·l},

where *ξ*_*i*_ = (*π*/6) · ∑*ρ*_*j*_ · *σ*_*j*_^*i*^, *ρ*_*j*_ is the number density, in molecules per Å^3^, of the species *j*, and *σ*_*j*_ is the corresponding hard sphere diameter; *ξ*_3_ = (*π*/6) · ∑*ρ*_*j*_ · *σ*_*j*_^3^ represents the volume packing density of the hard sphere fluid mixture (i.e., the fraction of the liquid volume actually occupied by solvent and cosolvent molecules and/or ions). By setting *l* = 0, the formula becomes right for a spherical cavity of radius *a*; by considering only one component, (1) corresponds to that for a hard sphere fluid.

To perform classic SPT calculations at 30°C and 1 atm, the experimental densities of water [13], 996 g L^−1^, aqueous 2 M TPACl solution [9], 980 g L^−1^, and aqueous 1 M TPA_2_SO_4_ solution, 1050 g L^−1^, have been used (i.e., the density of aqueous 1 M TPA_2_SO_4_ solution has been estimated from PMV data [9, 14]; so calculations have been performed also for larger density values, 1070 g L^−1^ and 1090 g L^−1^, to test the robustness of the emerged scenario). All the density values are listed in the second column of Table 1. Another critical point is the assignment of the effective hard sphere diameter to molecules and ions. I have fixed *σ*(H_2_O) = 2.80 Å, which is close to the location of the first peak of the oxygen-oxygen radial distribution function of water [15]. For the ions, I have fixed (a) *σ*(TPA^+^) = 6.98 Å, which is the value determined by Masterton and coworkers [16]; (b) *σ*(Cl^−^) = 3.62 Å, which is the value determined by Pauling [17]; (c) *σ*(SO_4_^2−^) = 4.60 Å, which is the value suggested by Marcus for the sulphate ion [18].

## 3. Results and Discussion

Classic SPT-calculated values of Δ*G*_*c*_(N) = Δ*G*_*c*_ (sphere) and Δ*G*_*c*_(D) = Δ*G*_*c*_ (spherocylinder), at 30°C and 1 atm, in all the considered aqueous solutions, are reported in the fifth and sixth columns of Table 1. The numbers emphasize that the Δ*G*_*c*_ magnitude increases in the following order: Δ*G*_*c*_(2 M TPACl) < Δ*G*_*c*_(1 M  TPA_2_SO_4_, *ρ* = 1050 g L^−1^) < Δ*G*_*c*_ (H_2_O). This rank order is not in line with that of the values of the volume packing density: *ξ*_3_ = 0.3824 in water, 0.4511 in 2 M TPACL, and 0.4687 in 1 M TPA_2_SO_4_, *ρ* = 1050 g L^−1^. This result can be rationalized by recognizing that the Δ*G*_*c*_ magnitude depends on two geometric properties [19]: the volume packing density of the solution and the average effective hard sphere diameter 〈*σ*〉 = ∑*χ*_*i*_ · *σ*_*i*_, where the *χ*_*i*_ are the molar fractions of the various species. Keeping fixed *ξ*_3_, Δ*G*_*c*_ increases on decreasing 〈*σ*〉 because the void space is partitioned in smaller pieces and the probability of finding molecular-sized cavities decreases. Keeping fixed 〈*σ*〉, Δ*G*_*c*_ increases on augmenting *ξ*_3_ because the void space in the liquid decreases and the probability of finding a molecular-sized cavity decreases. Note that, in general, about fifty percent of the total liquid volume is empty, but the volume available for the creation of a spherical cavity of given radius is the very very small fraction of the total void volume whose dimensions allow the occurrence of such cavity [10, 19]. By combining the *ξ*_3_ values listed in the fourth column of Table 1 with the 〈*σ*〉 values listed in the last column of Table 1, it should be straightforward to rationalize the obtained Δ*G*_*c*_ trend.

Moreover, it is clear that the Δ*G*_*c*_ magnitude, by keeping the cavity *V*_*vdW*_ fixed, depends markedly upon the cavity shape: Δ*G*_*c*_(D) > Δ*G*_*c*_(N) in all cases, because the spherocylinder has a larger WASA than the sphere, and WASA is a right measure of the solvent-excluded volume effect caused by cavity creation [6]. The calculated ΔΔ*G*_*c*_ = Δ*G*_*c*_(D) − Δ*G*_*c*_(N) numbers, at 30°C and 1 atm, are ΔΔ*G*_*c*_ = 377 kJ mol^−1^ in water, 311 kJ mol^−1^ in 2 M TPACl, and 345 kJ mol^−1^ in 1 M TPA_2_SO_4_, *ρ* = 1050 g L^−1^. Since the ΔΔ*G*_*c*_ term is always stabilizing the N-state, the above numbers emphasize that the stability of the N-state decreases in both aqueous 2 M TPACl solution and 1 M TPA_2_SO_4_, *ρ* = 1050 g L^−1^, solution with respect to the neat water case. Experimental PMV values [9, 14], at room temperature, of TPA^+^ ion, 214 cm^3^ mol^−1^, and SO_4_^2−^ ion, 14 cm^3^ mol^−1^, indicate that the density estimate used to perform classic SPT calculations is physically reliable. It does appear that the TPA^+^ ions are so large in size and interact so weakly with water molecules, due to their very low charge density, to be able to counteract the substantial electrostrictive effect of sulphate ions on the water structure, so that the density does not increase significantly. The emerged scenario is in line with the *salting in* of hydrocarbons caused by tetrapropylammonium bromide [20].

The reversible work of cavity creation can be calculated from the occurrence probability, at equilibrium, of molecular-scale density fluctuations [21]. The latter depend upon the size of liquid molecules and the liquid density, whose magnitude is a consequence of the strength of intermolecular interactions [21, 22]. This means that the density of aqueous salt solutions plays a fundamental role for the strengthening-weakening of hydrophobic interactions and the stabilizing-destabilizing activity of the different salts [5, 19, 23], together with attractive energetic interactions between ions and protein surface groups. Since the density of the aqueous solution plays a fundamental role in determining the Δ*G*_*c*_ magnitude and I have not found in the literature an experimental value for the density of the aqueous 1 M TPA_2_SO_4_ solution, I have performed the same classic SPT calculations for two other and greater densities: 1070 g L^−1^ and 1090 g L^−1^ (see the last two lines of Table 1). The obtained ΔΔ*G*_*c*_ values, 365 and 387 kJ mol^−1^, prove to be slightly smaller and slightly larger, respectively, than the value obtained in neat water, indicating that aqueous 1 M TPA_2_SO_4_ solution does not provide a real stabilization of the N-state. Therefore it should be reliable to state that this salt has a destabilizing activity against the folded state of globular proteins.

The present analysis indicates that the statistical thermodynamic approach developed by me to rationalize the physicochemical grounds of the conformational stability of globular proteins works well without the need of *ad hoc* assumptions [5, 6]. The difference in solvent-excluded volume on passing from the D-state to the N-state is the driving force of the folding process and is the main stabilizing factor. The magnitude of the solvent-excluded volume effect is affected by the addition, to water, of salts, osmolytes, or cosolvents because the latter significantly modify the water density and their molecules are markedly larger than that of water. Work is in progress in my lab to obtain experimental data on the density of aqueous TPA_2_SO_4_ solutions and to study the conformational stability of some small globular proteins in aqueous solutions of TPACl and TPA_2_SO_4_.

## Figures and Tables

**Table 1:** Experimental values, at 30°C and 1 atm, of the density and water molar concentration for neat water, aqueous 2 M TPACl solution, and aqueous 1 M TPA_2_SO_4_ solution (for the latter the values are estimates due to the lack of an experimental datum); values of the volume packing density for all these liquid solutions, calculated using the following effective hard sphere diameters: *σ*(H_2_O) = 2.80 Å, *σ*(TPA^+^) = 6.98 Å, *σ*(Cl^−^) = 3.62 Å, and *σ*(SO_4_^2−^) = 4.60 Å; classic SPT estimates of the reversible work to create in these liquid solutions, at 30°C and 1 atm, cavities corresponding to the N-state (i.e., a sphere of 10 Å radius) and to the D-state (i.e., a spherocylinder of 4 Å radius and 78 Å cylindrical length), respectively (these geometric values can be considered reliable for a 50-residue globular protein); values of ΔΔ*G*_c_ = Δ*G*_c_(D) − Δ*G*_*c*_(N) and values of the average effective hard sphere diameter 〈*σ*〉 of these liquid solutions.

	*ρ* g L^−1^	[H_2_O] M	*ξ*_3_	Δ*G*_c_(N) kJ mol^−1^	Δ*G*_c_(D) kJ mol^−1^	ΔΔG_c_ kJ mol^−1^	〈σ〉 Å
H_2_O	996	55.3	0.3824	495	872	377	2.80
2 M TPACl	980	29.9	0.4511	406	717	311	3.09
1 M TPA_2_SO_4_	1050	32.3	0.4687	452	797	345	3.09
1 M TPA_2_SO_4_	1070	33.4	0.4764	478	843	365	3.08
1 M TPA_2_SO_4_	1090	34.5	0.4841	507	894	387	3.07
